# Genetic and epidemiological analyses of infection load and its relationship with psychiatric disorders

**DOI:** 10.1017/S0950268823000687

**Published:** 2023-05-18

**Authors:** Ron Nudel, David M. Hougaard, Thomas Werge, Michael E. Benros

**Affiliations:** 1 CORE-Copenhagen Research Centre for Mental Health, Mental Health Centre Copenhagen, Copenhagen University Hospital, Copenhagen, Denmark; 2 The Lundbeck Foundation Initiative for Integrative Psychiatric Research (iPSYCH), Aarhus, Denmark; 3 Center for Neonatal Screening, Department for Congenital Disorders, Statens Serum Institut, Copenhagen, Denmark; 4 Institute of Biological Psychiatry, Mental Health Centre Sct. Hans, Mental Health Services Copenhagen, Roskilde, Denmark; 5 Department of Clinical Medicine, Faculty of Health and Medical Sciences, University of Copenhagen, Copenhagen, Denmark; 6 Department of Immunology and Microbiology, Faculty of Health and Medical Sciences, University of Copenhagen, Copenhagen, Denmark

**Keywords:** Genetics, infectious disease, infectious disease epidemiology, psychiatric disorders, heritability, genetic correlation, GWAS

## Abstract

Severe infections and psychiatric disorders have a large impact on both society and the individual. Studies investigating these conditions and the links between them are therefore important. Most past studies have focused on binary phenotypes of particular infections or overall infection, thereby losing some information regarding susceptibility to infection as reflected in the number of specific infection types, or sites, which we term infection load. In this study we found that infection load was associated with increased risk for attention-deficit/hyperactivity disorder, autism spectrum disorder, bipolar disorder, depression, schizophrenia and overall psychiatric diagnosis. We obtained a modest but significant heritability for infection load (*h^2^* = 0.0221), and a high degree of genetic correlation between it and overall psychiatric diagnosis (*r_g_* = 0.4298). We also found evidence supporting a genetic causality for overall infection on overall psychiatric diagnosis. Our genome-wide association study for infection load identified 138 suggestive associations. Our study provides further evidence for genetic links between susceptibility to infection and psychiatric disorders, and suggests that a higher infection load may have a cumulative association with psychiatric disorders, beyond what has been described for individual infections.

## Introduction

Infections are one of the major global health concerns and a leading cause of early mortality [1, 2]. In addition to the health risk conferred directly by severe infections, studies have shown them to be associated with an increased risk of mental disorders, including schizophrenia [3], mood disorders such as depression [4], and neurodevelopmental disorders such as autism spectrum disorder (ASD) [5]. While infections are inherently caused by external pathogens, susceptibility to infection has a genetic component [6].

In addition to replicating the epidemiological associations between psychiatric disorders and infections, we showed in a previous study that susceptibility to severe infections (i.e., infections requiring hospital contact) had a modest but significant heritability (3.2% on the observed scale) and that the genetic correlation between overall infection (defined as having at least one diagnosis from a variety of infection categories included in the study) and overall psychiatric diagnosis (International Classification of Diseases (ICD-10) codes F00–F99 and/or ICD-8 codes 290–315, but mostly one or more of schizophrenia, bipolar disorder or depression (affective disorder), ASD, attention-deficit/hyperactivity disorder (ADHD), and anorexia) was 0.4–0.5, depending on the method used to estimate it [7]. Another study that used the iPSYCH sample found genetic overlaps between susceptibility to infections and specific psychiatric disorders, namely schizophrenia, ADHD, depression, bipolar disorder, and post-traumatic stress disorder, but not ASD, using genetic correlation analysis and/or polygenic risk scores (PRSs) [8]. Interestingly, a recent study has shown positive genetic correlations between blood levels of C-reactive protein, a marker for infection and inflammation, and major depressive disorder (MDD) and ADHD, as well as positive genetic correlations between white blood cell counts and MDD, ADHD, and schizophrenia [9], which could provide insights into the molecular mechanisms underlying the genetic correlation between susceptibility to infection and psychiatric disorders, although the trends were not always similar across individual psychiatric disorders in that study.

The aim of this study was to investigate these links further by employing a quantitative phenotype for infection load in the iPSYCH cohort, comprising 65,534 individuals selected as cases of major psychiatric disorders (schizophrenia, bipolar disorder or depression (affective disorder), ASD, ADHD, and anorexia) or as part of a random population sample. Infection load in this study is defined as the number of site-specific infection categories (central nervous system infection, gastrointestinal infections, genital infections, hepatitis, otitis, pregnancy-related infections, respiratory infections, sepsis, skin infections, HIV/AIDS, and urological infections) for which an individual received at least one diagnosis. Thus, infection load in this sense makes use of register-based diagnoses, similar to other studies [10], to get a quantitative measure for an individual’s overall susceptibility to severe infections using binary trait data (the presence or absence of a diagnosis). This extends our previous study on overall infection as a binary trait. Additionally, we replicate our previous results and results from this study using an external sample and perform genetic correlation analyses both within and across the two samples.

## Methods

### Data sources for diagnoses and study sample

The sample and the phenotypes used in this study have been described in our previous publications [7, 11–15], and we repeat the details here, with changes relevant to the present study: the iPSYCH Consortium linked data from the Danish medical registers to biobank data via the unique civil registration number used in Denmark since 1968 [16]. Biological data from the Danish Neonatal Screening Biobank include dried blood spots taken 4–7 days after birth from nearly all infants born in Denmark after 1981 [16, 17], which were used for downstream genotyping. Infection diagnoses were obtained from the Danish National Hospital Register, which, since 1977, has included records of all inpatients treated in Danish non-psychiatric hospitals, and, since 1995, has included information regarding outpatient and emergency room contacts [18]. The Psychiatric Central Research Register includes data from all psychiatric inpatient facilities since 1969 and outpatient contacts since 1995 [19]. In Denmark, diagnoses were based on the 8th Revision of the ICD-8 [20] from 1977 to 1993, and, since 1994, they have been based on ICD-10 [21]. For the psychiatric phenotypes included in this study, all diagnosis types apart from henvisningsdiagnose (referral diagnosis) were included, and the types of contact included were inpatient, outpatient, and emergency care unit contact. Data for psychiatric phenotypes are from the Psychiatric Central Research Register. For infection phenotypes, the included types of diagnosis were the following: ICD-8: hoveddiagnose and bidiagnose (main and auxiliary diagnosis, respectively); ICD-10: aktionsdiagnose, grundmorbus, and bidiagnose (main, basic, and auxiliary diagnosis, respectively), and the included types of contact were inpatient and outpatient hospitalisations and emergency room contact, all from the Danish National Hospital Register. Tillægsdiagnoser (associated diagnoses) were not considered, and diagnoses of the following types were excluded: henvisningsdiagnose (referral) and komplikation (complication). ICD-8 diagnoses with the following modifications: ‘Obs. Pro’ and ‘Ej befundet’ (suspected and not found, respectively) were also excluded. All individuals in this study are part of the iPSYCH2012 sample [22], selected from among all individuals born in Denmark between 1981 and 2005 (*N* = 1,472,762), and which included individuals diagnosed with at least one of schizophrenia, bipolar disorder or depression (affective disorder), ASD, ADHD, and anorexia, and individuals who were selected as part of a randomly selected sample from the Danish population. The iPSYCH sample used in this study has undergone several rounds of quality control (QC) as described in our previous studies [7, 11–15] using data from high-quality genetic markers prior to the imputation. The main sample QC steps included the removal of individuals based on ancestry (individuals who did not have Danish ancestry, as determined from register data of family history and genetic principal component analyses had been removed), as well as relatedness (if they were first- or second-degree relatives of other individuals in the sample; this step prioritised iPSYCH cases and then individuals with a higher genotype call rate). Other QC steps involved the removal of individuals based on missingness (>1%), abnormal heterozygosity, ambiguous sex (discrepancies between annotated sex and genetic data), or if they were duplicates of other individuals. The first studies employing this QC protocol have more information about the procedures [23, 24], including the supplementary methods of a preprint of the former [25] and another study [26]. Before QC, 78,050 individuals from 23 genotyping waves were included. Following QC, 65,534 unrelated Danish individuals were retained, of whom 34,705 were male and 30,829 were female. Data up to the end of 2012 were included for infections, and data up to the end of 2013 were included for psychiatric diagnoses. We had diagnoses for the following infection categories: bacterial, viral, central nervous system infection, gastrointestinal, genital, hepatitis, otitis, pregnancy infection, respiratory, sepsis, skin infection, HIV/AIDS, urological, and other infections (e.g., protozoan infections). ICD codes for these are provided in Supplementary Table S1. For psychiatric disorders, the following ICD codes were used: any/overall psychiatric diagnosis (ICD-8 code within the range 290–315 and/or ICD-10 code within the range F00–F99); ADHD (ICD-10: F90.0); anorexia (ICD-8: 306.50; ICD-10: F50.0); ASD (ICD-10: F84.0, F84.1, F84.5, F84.8, and F84.9); bipolar disorder (ICD-8: 296.19, 296.39, and 298.19; ICD-10: F30 and F31); depression (single and recurring) (ICD-8: 296.09, 296.29, 298.09, and 300.49; ICD-10: F32 and F33); schizophrenia (ICD-8: 295.x9 (excl. 295.79); ICD-10: F20).

### Defining phenotypes for infection load and psychiatric diagnoses

For specific psychiatric disorders, case (control) status was determined based on having (not having) the relevant ICD diagnosis as per the above codes. For any/overall psychiatric disorder, cases had any ICD code from ICD-8: 290–315 and/or ICD-10: F00–F99, and controls did not have any of the codes from those ranges. For the quantitative phenotype of infection load, individuals who did not have any diagnosis from the above 14 infection categories received an infection load phenotype value of 0; for individuals with infection diagnoses, we counted the number of infection categories they had excluding the broad categories of bacterial, viral, and other infections, and the total count was used as their infection load value. This approach was chosen because these broad categories include codes that were also found in specific categories, and, moreover, they do not indicate specific infection sites. Individuals who were cases only for those three categories without any site-specific category (*N* = 3,074) received a missing infection load value and were not used in analyses for this phenotype. The final sample used in this study, therefore, included 62,460 individuals for the infection load phenotype (*NB:* when using the binary overall infection phenotype in some analyses, the full sample was used). The phenotype distribution figure was exported using Daniel’s XL Toolbox v7.3.4 [27].

### Statistical and epidemiological analyses

Statistical analyses were performed in R [28] v.3.5.1. Logistic regressions of psychiatric diagnosis on infection load were performed with the *glm* function (family = binomial(link = ‘logit’)) with covariates for age (in years), age squared, and sex, resulting in two-sided *p*-values from a Wald test for the infection load coefficient’s being different from zero. Confidence intervals were calculated with the *confint* function. The regressions were performed in the random population sample (*N* = 21,706 before exclusions; *N* = 20,822 after exclusions for the infection load phenotype) to avoid potential biases and obtain population estimates, and controls for psychiatric disorders were ‘normal controls’, that is, they did not have the diagnosis which was being tested but they could have other (psychiatric) diagnoses. Note that the age covariates in this study were censored for a minority of individuals who emigrated (0.43%), died (1.02%), or lost contact with the Danish authorities (0.02%) by July 2013 (i.e., we set their age to what it was when their status changed, whereas, for other individuals, the age at the end of 2013 was used). These figures apply to the full sample. Tetrachoric correlations for overall infection and overall psychiatric diagnosis in the full sample and in the random population sample were calculated with the polycor R package v.0.8-1 [29].

### Genetic dataset and genome-wide association study for infection load

We performed a discovery genome-wide association study (GWAS) for infection load, and GWASs for other phenotypes (overall psychiatric diagnosis; overall infection) for downstream heritability and genetic correlation analyses. The description of the genetic dataset used in this study has appeared in several of our previous publications [11, 12], but we repeat it briefly here, with further information relevant to the present study: the marker dataset used in this GWAS has undergone several rounds of QC. The starting point for the QC was a dataset which had 78,050 samples genotyped in 23 of the original 25 waves. This dataset is described in detail elsewhere [30]. Further QC of this dataset including a description of the procedures for sample and marker QC is provided in the first study that utilised it [23]. Note, however, that that study had a minor allele frequency (MAF) threshold (for dosage data) different from the one below. Before the imputation of more markers, markers with rare alleles and non-autosomal markers were removed. The pre-imputation QC is described in more detail in other studies which used this sample [23, 24, 26]. For the imputation, genotypes were phased with SHAPEIT3 [31] and the imputation was performed with IMPUTE2 [32]. Following the imputation, markers were excluded if they had an INFO score (calculated with QCTOOL) <0.2; MAF <0.001; best-guess genotypes missing in >10% of subjects, whereby the missingness in imputed genotypes was determined by treating individual genotypes with probability <0.9 as missing; Hardy–Weinberg equilibrium *P* < 1 × 10^−6^; and/or a genome-wide significant association with the genotyping wave or with the imputation batch itself (in controls in a homogeneous European subset of the sample). Lastly, markers with differential missingness between psychiatric cases and controls (*P* < 1 × 10^−6^) were also removed. In this study, we used only best-guess genotypes, or hard calls, and only genotypes with a probability of 0.9 or above (i.e., the imputation resulted in a best-guess genotype with a probability of at least 0.9 for a given marker and a given individual) were retained (others were set to missing). Furthermore, markers were retained only if they had an INFO score (following the imputation) of at least 0.8 and MAF of at least 0.01 in the QCed sample (these steps were additional steps not included in some of the previous studies mentioned [23, 24, 26]). Ultimately, 7,071,055 markers were used in the GWASs. The genome build for our dataset was hg19. The infection load discovery GWAS was performed with PLINK v.1.90b6.18 using a linear regression model (*--linear*) and included covariates for age, age squared, sex, the first 10 principal components, and overall psychiatric diagnosis status. The Manhattan plot and the QQ plot were generated with the ‘qqman’ R scripts by Stephen Turner and Daniel Capurso (with the (major update) version from 19 April 2011 for the former plot and the version from 10 June 2013 for the latter, available at https://github.com/stephenturner/qqman/blob/v0.0.0/qqman.r). Other GWASs (with logistic regression in the case of binary phenotypes, with *--logistic*) were performed for downstream analysis with LDSC, as described below. Those GWASs included covariates for age, age squared, sex, and the first 10 principal components. A covariate for overall psychiatric diagnosis status was included in some analyses for the heritability estimates and not in others (as indicated in the Results section), and it was not included in GWASs used in downstream genetic correlation analyses. For the top markers, a post hoc association test was run in R using a Poisson regression with the *glm* function (family = poisson(link = ‘log’)) and the same covariates as in the GWAS, as the infection load phenotype consisted of counts and was not normally distributed.

### Heritability estimates and genetic correlations

Using the same dataset of markers and covariates as the ones used in the GWAS, we estimated the heritability of infection load in our full sample. This was achieved with GCTA [33] as well as LDSC [34, 35]. For GCTA, the genetic relationship matrix (GRM) was calculated for each autosomal chromosome separately with *--make-grm* and merged with *--mgrm* with GCTA v1.91.1 beta as previously described [14]. The heritability of infection load was estimated with *--reml* in GCTA v1.93.2 beta (covariates included age, age squared, sex, the first 10 principal components, and overall psychiatric diagnosis status). The number of markers in the final GRM for the full sample was 6,941,439. For LDSC, LD score files were generated using the QC-passing random population sample (from the marker dataset with best-guess genotype hard calls updated to have genetic positions using the genetic map from 1000 Genomes phase 3), with a 1 cm window (*--l2 --ld-wind-cm 1*), as described previously [7]. The summary statistics (PLINK output) from the GWAS were processed with the *munge_sumstats.py* script with the default parameters (after adding A2 from the PLINK bim file and using the NMISS column as the *N* for LDSC), and LDSC (*ldsc.py*) v.1.0.1 was used (with the default parameters) in the estimation of the heritabilities (with *--h2*) and genetic correlations (with *--rg*). We used the same LD score dataset as both reference and regression (*--ref-ld-chr* and *--w-ld-chr*) datasets, as recommended in the LDSC tutorial for non-partitioned LD Score regression (https://github.com/bulik/ldsc/wiki/Heritability-and-Genetic-Correlation, version from 13 July 2017). In the iPSYCH datasets, 5,464,859 markers remained after processing with *munge_sumstats.py.* We tested the heritability (*h*^2^) as being different from zero using a Wald test and the *χ*^2^ distribution with 1 degree of freedom (d.f.) in the following way: *χ*^2^ = (*h*^2^/SE)^2^, where *h*^2^ is the heritability on the observed scale from LDSC and SE is the standard error for the heritability, and *p*-values were calculated with 0.5*pchisq(χ^2^, df = 1, lower.tail = F) in R (we multiply by 0.5 since *h*^2^ should be nonnegative). For GCTA, the likelihood ratio test statistic from the GCTA output was used to derive *p*-values using the *χ*^2^ distribution with 1d.f., as described above for the LDSC *h*^2^ estimates. Genetic correlations were estimated only with LDSC, as it is robust to sample overlap for the studied traits and allowed the use of only summary statistics-level data from the replication sample. Note that when testing for genetic correlations between infection phenotypes and psychiatric disorders in iPSYCH, the summary statistics for the infection phenotypes in iPSYCH were from a GWAS that did not include a covariate for overall psychiatric diagnosis status, as stated previously. For heritability estimation with LDSC, a covariate for overall psychiatric diagnosis status was included in the corresponding GWAS, or not, as indicated in the Results section. All GWASs used in downstream LDSC analyses included covariates for age, age squared, sex, and the first 10 principal components. The genetic correlation (*r_g_*) was tested for being different from zero using a Wald test as well: *χ*^2^ = (*r_g_*/SE)^2^, where *r_g_* is the genetic correlation from LDSC and SE is the standard error for the genetic correlation. *P*-values were calculated in R using *pchisq(χ^2^, df = 1, lower.tail = F).* Note that in the case of the heritabilities and genetic correlation for or between any infection and any psychiatric diagnosis, which we reported previously [7], we include in this study results that used the censored age covariates, so as to be in line with the present study’s methodology. For the binary traits, heritabilities were transformed to the liability scale [36] using the proportion of cases in each GWAS and a population prevalence estimate from the iPSYCH random population sample (0.1 for overall psychiatric diagnosis and 0.357 for overall infection).

### Replication sample

To replicate the results from the present study and our previous study with a binary overall infection phenotype, we used summary statistics from FinnGen [37] Release 7 (309,154 individuals). We used two phenotypes which were the closest possible to our any/overall infection and any/overall psychiatric diagnosis phenotypes: certain infectious and parasitic diseases (AB1_INFECTIONS, based on ICD-10 codes starting with A or B) and any mental disorder (KRA_PSY_ANYMENTAL, based on ICD-10 codes F00–F03, F051, G30, and F1–F9, or ICD-8/9 equivalent codes) with the following numbers of cases and controls: 86,892 and 222,262, and 76,073 and 233,081, respectively. The FinnGen summary statistics were processed in the following way: first, they were cross-referenced with the HRC reference panel (ftp://ngs.sanger.ac.uk/production/hrc/HRC.r1-1/HRC.r1-1.GRCh37.wgs.mac5.sites.tab.gz) based on the marker ID. From that, the chromosome and position in genome build hg19 were added to the summary statistics. Based on the chromosome and position in hg19, marker IDs were changed to corresponding marker IDs in the iPSYCH dataset, and only overlapping markers were retained. This made it possible to use the same LD score files and to have greater marker overlap between the iPSYCH and FinnGen datasets for the genetic correlation analyses. Duplicate markers and markers with mismatched alleles (identified using PRSice [38] v2.3.5) were removed from the summary statistics. The QCed summary statistics were then processed with *munge_sumstats.py* (as they did not contain sample sizes per marker, the total sample size as per the above numbers was used). After QC and processing, 4,887,803 markers were included in the FinnGen datasets. It should be noted that our LD score files were based on a homogenous Danish-European sample; while there exist LD differences between Finns and Danes, studies have used the same (European) LD weights when analysing both Finnish and Danish samples for genetic correlation estimation with LDSC [39]. However, we also checked the heritabilities and genetic correlations within FinnGen using European LD scores (https://data.broadinstitute.org/alkesgroup/LDSCORE/eur_w_ld_chr.tar.bz2). In these analyses, we did not convert marker IDs, as both FinnGen datasets and the LD score files included SNP rsIDs. Preprocessing with *munge_sumstats.py* in this case included adding the total sample size as per the above; otherwise, the default thresholds we used. After processing, 12,809,821 markers in each dataset (AB1_INFECTIONS and KRA_PSY_ANYMENTAL) were retained (excluding markers with no rsID in the summary statistics files). The number of overlapping markers across the FinnGen datasets and the European LD score dataset was 1,169,856. Heritabilities were transformed to the liability scale using the proportion of cases for each trait as reported by FinnGen, and the same values were used for the population prevalence (since FinnGen is a full population cohort).

### Post hoc analyses for genetic correlations

We performed several post hoc analyses and sensitivity analyses to evaluate whether various factors affected the genetic correlation analyses and/or provide further insight into the genetic relationship between infection and mental illness. The first of these was a test for cross-trait assortative mating, which could influence the *r_g_* estimate. It was shown that, for a single trait, assortative mating would induce a correlation between a PRS generated using only odd- or only even-numbered chromosomes (in the target sample) at a time and that, under random mating, these scores should not be correlated [40]. This approach was later extended to cross-trait assortative mating [41], whereby the PRS for trait 1 generated using only odd-numbered chromosomes is tested for correlation with the PRS for trait 2 generated using only even-numbered chromosomes, and vice versa. We used a simplified version of this procedure, testing for both correlations at both an inclusive *p*-value threshold (*pT* = 1) and the genome-wide significance threshold (*pT* = 5 × 10^−8^), with FinnGen summary statistics (as processed above for genetic correlations with iPSYCH) as the training dataset and iPSYCH as the target dataset. The PRSs were generated with PRSice v.2.3.5 with an *r*^2^ threshold of 0.1 in a window of 250 kbp and using the *--score sum* method. Otherwise, the default parameters were used. The correlation was tested with the *cor.test* function in R, using the Pearson correlation as the method. The tests were two-sided.

To test for genetic causality between overall infection and overall psychiatric diagnosis in iPSYCH, we used the latent causal variable (LCV) model [42]. This model tests whether trait 1 (in our case, overall infection) is causal to trait 2 (overall psychiatric diagnosis) using a latent causal variable which is assumed to mediate the genetic correlation between the two traits. If trait 1 is strongly genetically correlated with this variable, then it is partially genetically causal to trait 2. This relationship is quantified using the genetic causality proportion (GCP). GCP ranges from 0 (no genetic causality) to 1 (full genetic causality; if it is negative, it means that trait 2 is partially or fully genetically causal to trait 1). LCV uses LD scores and summary statistics (we used the same LD scores as used with LDSC, and the summary statistics processed with LDSC), as LDSC does, but it uses them in a modified procedure. We used both the full dataset of summary statistics and markers with MAF ≥ 0.05 as recommended on the LCV GitHub page. The scripts used were https://github.com/lukejoconnor/LCV/blob/master/R/ExampleRealdataScript.R, https://github.com/lukejoconnor/LCV/blob/master/R/MomentFunctions.R, and https://github.com/lukejoconnor/LCV/blob/master/R/RunLCV.R. The versions used were from 1 July 2020 for the first script and 14 March 2019 for the last two scripts.

Lastly, we performed sensitivity analyses for the LDSC results after removing the major histocompatibility complex (MHC) region on chromosome 6 from the dataset used to generate the LD scores: we removed markers on chromosome 6 between coordinates 25,392,021 and 33,392,022, based on the coordinates from a GWAS protocol [43] converted to genome build hg19 using the UCSC Genome Browser LiftOver tool, and regenerated the LD scores with LDSC v1.0.1 using the dataset of the random population sample (as described earlier). The LDSC analyses were then repeated using these new LD scores. The numbers of markers included in the *h*^2^ and *r_g_* analyses after the removal of the MHC region were 5,424,093 for the iPSYCH datasets and 4,857,454 for the FinnGen datasets (when used with iPSYCH LD scores). Note that the external European LD score dataset did not include the MHC region to begin with.

## Results

In the full iPSYCH sample, 24,161 individuals had a psychiatric diagnosis but no infection diagnosis, 6,744 had an infection diagnosis but no psychiatric diagnosis, 21,728 had both, and 12,901 had neither. In the random population sample, 1,113 had a psychiatric diagnosis but no infection diagnosis, 6,693 had an infection diagnosis but no psychiatric diagnosis, 1,062 had both, and 12,838 had neither. The tetrachoric correlation between overall infection and overall psychiatric diagnosis was 0.2006 (SE = 0.0063) in the full sample and 0.1923 (SE = 0.0143) in the random population sample. Sample sizes for psychiatric disorders and the binary infection phenotype are shown in Table 1. To examine the age relationship between the two disease classes, we used individuals who had both diagnoses and defined the following variable: age at (first) infection category diagnosis minus age at (first) psychiatric diagnosis (both in years). This variable had a mean of −8.45, a median of −8.21, and a standard deviation of 8.17. In the random population sample, these were −8.79, −8.89, and 8.24, respectively. This suggests that, on average, the first severe infection was diagnosed before the first psychiatric diagnosis in our sample. For infection load, the counts of individuals per infection load value are shown in Table 2. Among individuals with an infection load of 0 in the full sample, the proportion of individuals with overall psychiatric diagnosis was 65%, which rose to 85% for individuals with an infection load of 5 or above. Among individuals with an infection load of 0 in the random population sample, the proportion of individuals with overall psychiatric diagnosis was 8%, which rose to 27% for individuals with an infection load of 5 or above. Supplementary Figure S1 shows the proportion of individuals with any psychiatric diagnosis across infection load values.

**Table 1.** Sample sizes for psychiatric disorders and overall infection phenotype

Phenotype	Number of individuals in the full sample	Number of individuals in the random population sample
Full sample size	65,534	21,706
Overall infection	28,472	7,755
Overall psychiatric diagnosis	45,889	2,175
Autism spectrum disorder	12,331	277
Attention-deficit/hyperactivity disorder	14,397	338
Schizophrenia	2,401	70
Bipolar disorder	1,391	36
Depression	18,511	435
Anorexia	2,551	50

**Table 2.** Distribution of infection load in the full sample and in the random population sample

Infection load	Number of individuals in the full sample	Number of individuals in the random population sample
0	37,062	13,951
1	17,682	5,166
2	5,858	1,366
3	1,460	271
4	324	53
5–7^a^	74	15

a Due to the data information policy of the iPSYCH Consortium, exact numbers that are lower than 5 (excluding 0) are not specified, which is why we combined the counts for infection load 5–7; this was done only for reporting purposes, and the actual analyses used the individual counts.

### Comorbidity analyses for infection load and psychiatric disorders

Infection load was significantly associated with an increased risk of all individual psychiatric disorders except for anorexia. It was especially associated with overall psychiatric diagnosis, with an odds ratio (OR) of 1.4578 (*P* = 1.10 × 10^−41^). Table 3 shows the results of the regression analyses.

**Table 3.** Results of regressions of psychiatric disorder on infection load in the random population sample

Disorder	Estimate from the regression	SE (estimate)	*P*-value	OR	OR 95% CI lower bound	OR 95% CI upper bound
Autism spectrum disorder	0.3084	0.0750	3.94 × 10^−5^	1.3613	1.1698	1.5703
Attention-deficit/hyperactivity disorder	0.4250	0.0626	1.16 × 10^−11^	1.5297	1.3488	1.7247
Schizophrenia	0.4309	0.1241	0.000519	1.5386	1.1890	1.9375
Bipolar disorder	0.4548	0.1684	0.006911	1.5758	1.1038	2.1444
Depression	0.4102	0.0530	9.96 × 10^−15^	1.5072	1.3560	1.6694
Anorexia	0.1325	0.1915	0.488794	1.1417	0.7584	1.6132
Overall psychiatric diagnosis	0.3770	0.0279	1.10 × 10^−41^	1.4578	1.3800	1.5393

*Note*: The *p*-values are not corrected. CI, confidence interval; OR, odds ratio.

### Heritabilities and genetic correlations

Infection load showed a significant nonzero heritability, but it was not high, at *h*^2^ = 0.0221 (SE = 0.0055, *P* = 2.93 × 10^−5^) from LDSC. The heritability estimate for infection load from GCTA was similar: *h*^2^ = 0.0432 (SE = 0.0053, LRT statistic = 77.298, *P* = 7.35 × 10^−19^). GCTA used a genomic relationship matrix which was based on more markers than included in the summary statistics used by LDSC, which could contribute to the difference between these estimates, in addition to the methodological differences between GCTA and LDSC. The binary overall infection and overall psychiatric diagnosis phenotypes in iPSYCH and the corresponding phenotypes in FinnGen were also significantly different from zero, with the psychiatric phenotypes showing higher heritabilities. Table 4 shows the results of all LDSC heritability analyses.

**Table 4.** Heritability estimates from LDSC

Trait	Observed scale *h*^2^	SE	*P*-value	Liability scale *h*^2^
iPSYCH infection load (with covariate for overall psychiatric diagnosis)	0.0221	0.0055	2.93 × 10^−5^	–
iPSYCH infection load (without covariate for overall psychiatric diagnosis)	0.0252	0.0055	2.30 × 10^−6^	–
iPSYCH overall infection (with covariate for overall psychiatric diagnosis)	0.0157	0.0048	0.0005362	0.0242
iPSYCH overall infection (without covariate for overall psychiatric diagnosis)	0.0178	0.0048	0.0001043	0.0274
iPSYCH overall psychiatric diagnosis	0.0763	0.0061	3.37 × 10^−36^	0.0956
FinnGen infections (iPSYCH LD scores)	0.0118	0.0030	4.19 × 10^−5^	0.0210
FinnGen infections (European LD scores)	0.0159	0.0020	9.33 × 10^−16^	0.0283
FinnGen any mental disorder (iPSYCH LD scores)	0.0260	0.0019	6.31 × 10^−43^	0.0486
FinnGen any mental disorder (European LD scores)	0.0437	0.0028	3.25 × 10^−55^	0.0817

*Note*: The *p*-values are not corrected. ‘Liability scale *h*^2^’ is relevant only to binary traits; in all cases, the *h*^2^ in the ‘observed scale *h*^2^’ column is the one reported by LDSC. SE, standard error.

For the binary phenotypes, our genetic correlation analyses replicated our previous results. Namely, overall infection was highly correlated with overall psychiatric diagnosis within iPSYCH, within FinnGen, and across FinnGen and iPSYCH, with estimates mostly being higher than 0.4. For infection load, the genetic correlation with overall psychiatric diagnosis was slightly higher than that between overall infection and overall psychiatric diagnosis. For the corresponding phenotypes across iPSYCH and FinnGen, the genetic correlations were above 0.6. All estimates remained significant after the Bonferroni correction for the number of genetic correlations estimated in this study. The full results are shown in Table 5.

**Table 5.** Genetic correlation estimates from LDSC

Trait 1	Trait 2	*r_g_*	SE	*P*-value
iPSYCH overall infection	iPSYCH overall psychiatric diagnosis	0.4061	0.1049	0.000108
iPSYCH overall infection	FinnGen any mental disorder	0.4131	0.0956	1.55 × 10^−5^
iPSYCH overall infection	FinnGen infections	0.6189	0.1394	9.01 × 10^−6^
iPSYCH overall psychiatric diagnosis	FinnGen any mental disorder	0.6660	0.0444	7.34 × 10^−51^
iPSYCH overall psychiatric diagnosis	FinnGen infections	0.4062	0.1118	0.000280
iPSYCH infection load	iPSYCH overall psychiatric diagnosis	0.4298	0.0901	1.84 × 10^−6^
iPSYCH infection load	FinnGen infections	0.6347	0.1279	6.96 × 10^−7^
iPSYCH infection load	FinnGen any mental disorder	0.3388	0.0733	3.80 × 10^−6^
iPSYCH infection load	iPSYCH overall infection	0.9837	0.0364	7.57 × 10^−161^
FinnGen infections (iPSYCH LD scores)	FinnGen any mental disorder (iPSYCH LD scores)	0.5603	0.1110	4.47 × 10^−7^
FinnGen infections (European LD scores)	FinnGen any mental disorder (European LD scores)	0.6465	0.0555	2.33 × 10^−31^

*Note*: When at least one of the traits was measured in iPSYCH, iPSYCH-generated LD scores were used. The *p*-values are not corrected. SE, standard error.

### Top results from the discovery GWAS for infection load

**Figure 1.**
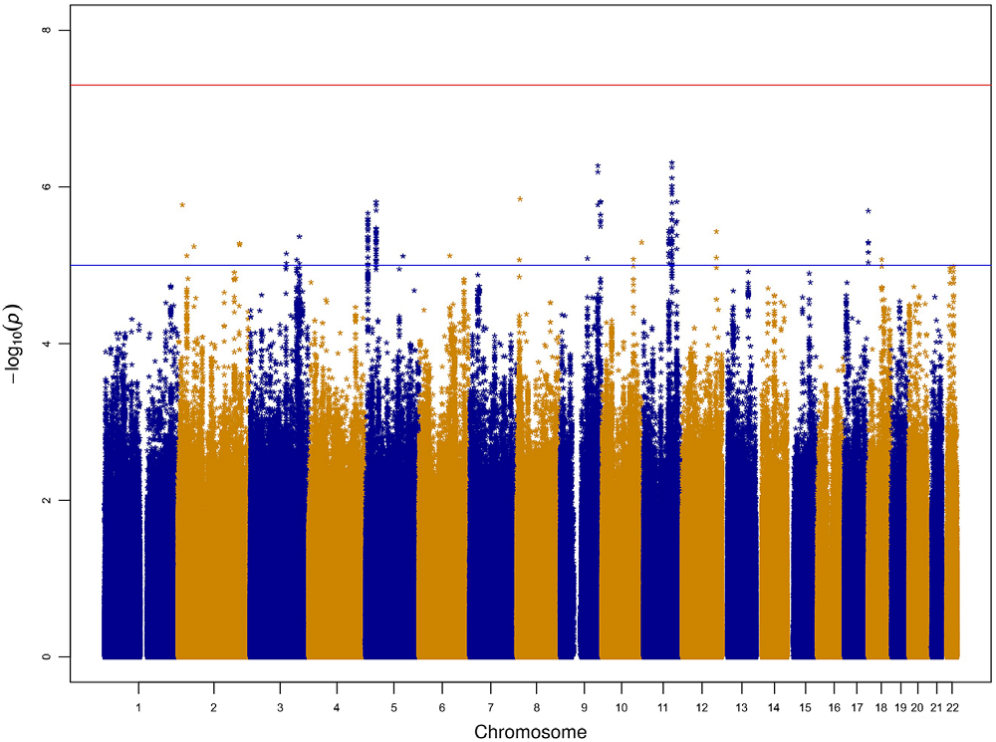
Manhattan plot for the genome-wide association study for infection load. The blue line represents the threshold for suggestive association (*P* = 1 × 10^−5^), and the red line represents the threshold for genome-wide significance (*P* = 5 × 10^−8^).

Our GWAS found 138 associations at the suggestive threshold (1 × 10^−5^), which are shown in Supplementary Table S2. Figure 1 shows the Manhattan plot for the GWAS, and the corresponding QQ plot is shown in Supplementary Figure S2. The top association in our study was with marker rs12361013 on chromosome 11 (*β* = −0.0323, *P* = 4.88 × 10^−7^, effect allele: A, other allele: G), which was the leading SNP in the suggestive association peak on chromosome 11. There was also a second suggestive association peak, almost equally significant, on chromosome 9, with the leading SNP being rs35711908 (*β* = 0.0242, *P* = 5.33 × 10^−7^, effect allele: A, other allele: G). These two markers were manually tested in a Poisson regression in R, due to the fact that the phenotype consisted of count data. This type of regression model is not implemented in common genetic association tools, but it may be more suitable for the infection load phenotype. For rs12361013, we obtained an estimate (beta) of −0.05848 for the A allele count, with *P* = 7.64 × 10^−8^. For rs35711908, we obtained an estimate of 0.04231 for the A allele count, with *P* = 1.14 × 10^−7^. Thus, at least for the top results, the linear regression analysis in PLINK underestimated the effects, but the differences were not large.

### Tests for assortative mating and causal relationship between overall infection and overall psychiatric diagnosis, and sensitivity analyses for genetic correlations

All cross-trait PRS correlations between odd- and even-numbered chromosomes were close to zero and nonsignificant: for infection PRS for odd-numbered chromosomes and psychiatric diagnosis PRS for even-numbered chromosomes, the correlations were −0.0018 (*P* = 0.6388) and 0.0042 (*P* = 0.2875) for *pT* = 1 and *pT* = 5 × 10^−8^, respectively. For infection PRS for even-numbered chromosomes and psychiatric diagnosis PRS for odd-numbered chromosomes, the correlations were 0.0002 (*P* = 0.9560) and 0.0044 (*P* = 0.2643) for those two *p*-value thresholds.

The LCV analyses showed some evidence for a causal relationship between overall infection and overall psychiatric diagnosis with GCP = 0.71 (*P* = 6.41 × 10^−5^) with all markers and GCP = 0.77 (*P* = 4.65 × 10^−8^) using markers with MAF ≥ 0.05. The LCV *Z*-scores for the heritability of overall infection were 5.96 and 5.59, respectively. The *h*^2^ *Z*-scores for overall psychiatric diagnosis were >7.

Our LDSC sensitivity analyses after the removal of the MHC region obtained results that were similar to the original results in terms of the sizes of the correlations, with the average change across all *h*^2^ estimates being ~0.001 and the average change across all *r_g_* estimates being ~0.051. The full results can be found in the Supplementary Notes for this paper.

## Discussion

Our study identified strong epidemiological and genetic associations between infection load and psychiatric disorders. In both cases, the association was positive, that is, an OR greater than 1 in a regression of a psychiatric diagnosis on infection load or a positive genetic correlation. Our previous study obtained higher ORs for the effect of the (binary) overall infection phenotype [7] on psychiatric diagnosis (it will be noted that the results of our previous study do not change much after censoring the age for individuals who died, emigrated or lost contact with the Danish authorities, as employed in the present study. For example, for overall psychiatric diagnosis, before age-censoring, the OR for overall infection was 1.74 (95%CI: 1.59-1.90), and after age censoring it was 1.73 (95%CI: 1.58-1.90)). However, in this study, we report the increase in odds per infection category/site on psychiatric diagnosis, in this study, we report the increase in odds per infection category/site (Table 3). Thus, having been diagnosed with, for example, infections of five different categories would increase the odds of having a psychiatric diagnosis by a factor of 6.6. In our sample, individuals tended to have been diagnosed with the (first) infection before the (first) psychiatric diagnosis. When looking at the quantitative phenotype of infection load, we observe a general positive association trend between infection load and the proportion of psychiatric cases (Supplementary Figure S1). While the temporal relationship between the two disease classes is obscured with the infection load phenotype, we know from the literature that some somatic diseases have a bidirectional relationship with psychiatric disorders and that the latter are also associated with the risk of somatic diseases even a decade later [44, 45]. Infections can also have a bidirectional relationship with psychiatric disorders [46].

The heritability of infection load is similar to the heritability of overall infection on the liability scale, and, as expected, they are strongly correlated genetically. Infection load shows a slightly higher genetic correlation (than overall infection) with overall psychiatric diagnosis in iPSYCH but also a lower one with FinnGen; however, in all cases, the genetic correlation is positive and remains significant after the Bonferroni correction. The genetic correlation between overall infection and overall psychiatric diagnosis replicated between iPSYCH and FinnGen (using infections in iPSYCH and psychiatric diagnosis in FinnGen and vice versa) and was about 0.4. It should be noted that the genetic correlations between the corresponding phenotypes across iPSYCH and FinnGen were not 1, but they were >0.6. This is not uncommon when the phenotypes were ascertained differently and come from different studies (e.g., as in the case of ASD in iPSYCH and in PGC [30]), and in our case, there were differences in sample composition and (mostly for infections) differences in the included diagnoses. The highest genetic correlation between overall infection and overall psychiatric diagnosis was obtained when using FinnGen summary statistics for both phenotypes, with European LD scores. Combined, the results of all analyses suggest that there is a high degree of positive genetic correlation between susceptibility to severe infections and psychiatric disorders. We found no evidence that the genetic correlation between overall infection and overall psychiatric diagnosis was inflated by potential cross-trait assortative mating between susceptibility to infection and psychiatric disorders. We found some evidence that susceptibility to overall infection is partially genetically causal to overall psychiatric diagnosis using the LCV model. We note that the LCV *Z*-scores for the heritability of overall infection were below 7, the threshold recommended in the LCV script; however, the LCV script also specifies that it is ‘a very stringent threshold’. These results do not mean that specific psychiatric disorders are necessarily linked to infections via genetics in the same way as overall psychiatric diagnosis. For example, we have shown in a previous study that susceptibility to infection is not genetically correlated with ASD, but genetics does play a role in the interplay between infections and ASD [11]. Namely, ASD cases with a history of maternal pregnancy-related infections were genetically different, as a group, from ASD cases without a history of maternal infections (the genetic correlation between the two phenotypes was *r_g_* = 0.3811, *P* = 0.0033 when testing against a null of 0, *P* = 1.89 × 10^−6^ when testing against a null of 1). ASD cases with a history of maternal infections occurring more than 2 months following birth were not genetically distinct from the group of ASD cases without a history of maternal infections (i.e., their genetic correlation was not significantly different from 1). These estimates do not change much when the MHC region is excluded from the analysis (discussed in more detail below). As ASD cases form a large part of the iPSYCH case subset, this may suggest that the underlying factors in the high genetic correlation between overall infection and overall psychiatric diagnosis could be non-disorder-specific with regard to psychiatric disorders or that they are driven by other disorders in iPSYCH. Lastly, our LDSC analyses included markers in the MHC region. Most studies that use LDSC remove these due to the high level of LD in the MHC region because such markers could be outliers in the regression model and influence the LDSC regression. However, in the iPSYCH GWASs in this study and our previous studies [7, 11], there was no strong signal in the MHC region (for infection phenotypes, this was true both for GWASs with and without a covariate for overall psychiatric diagnosis). The problem with LDSC and the MHC region having extremely strong associations pertains mostly to autoimmune diseases [47], and we did not have any such signal in our results. Also, given the relevance of the MHC to infection-related phenotypes, we decided to retain it in the analyses. However, we also provide results for the LDSC analyses repeated after removing the MHC region from the LD score datasets, for *h*^2^ and *r_g_* estimates reported in this study or relevant studies that employed the iPSYCH-generated LD scores. These can be found in the Supplementary Notes. The removal of the MHC region affected mostly analyses involving the FinnGen infections phenotype (when using the iPSYCH LD scores). This is likely due to the fact that, unlike the iPSYCH overall infection GWAS, the FinnGen infection GWAS did obtain a genome-wide significant signal within the MHC region, and, therefore, the analyses without the MHC region could be more appropriate when this dataset is used. A previous study that used the iPSYCH sample examined genetic correlations between infections and specific psychiatric disorders, and positive genetic correlations were found between infections and anorexia, ADHD, bipolar disorder, depression, and schizophrenia [8].

Our GWAS for infection load identified two suggestive association peaks. Interestingly, the top association from our GWAS for overall infection, with rs6447952, was not among the suggestive associations for infection load. In this context, one important point to consider is what our phenotype of infection load captures. By definition, all individuals with infection load >0 would be cases for our overall infection phenotype, and, therefore, we expect some overlap in genetic associations, as also reflected in the high genetic correlation between the phenotypes (which, it should be noted, could be very high in part due to the low heritabilities of the individual phenotypes). However, a high infection load, as defined in our study, suggests a high predisposition to infection regardless of the site of infection. In other words, it may indicate a deeper cause of immune dysfunction. This is not the same as simply having an infection or even being prone to a specific type of infection: an individual with 20 skin infections of the same kind and no other infection would have an infection load of 1, whereas an individual with a skin infection, a gastrointestinal infection, and a central nervous system infection would have an infection load of 3, and both individuals would have the same affection status for overall infection. This also makes biological sense: repeated infections of the same type may suggest a narrower genetic risk, or a primarily non-genetic risk factor, for example, personal hygiene in the case of skin infection, or diet in the case of gastrointestinal infection, whereas an individual with multiple infections at different sites of the body is likely to have a more general immune deficit. Thus, there could be genetic factors that might be captured by one phenotype but not the other.

Our top GWAS association was with rs12361013, which is located in the Contactin 5 (*CNTN5*) gene on chromosome 11. Contactin 5 is an immunoglobulin cell adhesion molecule which is involved in neural development and has been implicated in ASD [48]. It has also been implicated in inflammatory diseases such as gout [49] and in the response to anti-tumour necrosis factor alpha medication in Crohn’s disease patients [50]. Moreover, a variant in *CNTN5* showed a genome-wide significant association with secreted Interleukin-2 in response to vaccinia virus stimulation in individuals who had received a smallpox vaccine [51]. The second highest association was with rs35711908 on chromosome 9. This variant showed genome-wide significant associations with several phenotypes in FinnGen (Release 7, accessed through the web portal on 9 October 2022), including various forms of hypothyroidism and disorders of the thyroid gland, which, in turn, may be associated with infections [52, 53]. On the GTEx web portal (Version 8, accessed on 9 October 2022), rs35711908 is associated with the expression of the Proteasome 20S Subunit Beta 7 (*PSMB7*) and NIMA Related Kinase 6 (*NEK6*) genes in several tissues. *PSMB7* encodes a subunit of the 20S proteasome, which is responsible for protein degradation; upon stimulation by pro-inflammatory cytokines, this subunit is down-regulated and replaced by one encoded by *PSMB10*, which forms part of the immunoproteosome, which is involved in MHC class I antigen presentation [54, 55]. *NEK6* is involved in mitosis and, if overexpressed, may result in human B-cell lymphoma [56]. Interestingly, both *PSMB7* and *NEK6* interact or are predicted to interact with B-cell-specific enhancers [56, 57]. Thus, our top GWAS results do show some links to immune-related functions, but they are not genome-wide significant.

## Conclusions

Our study identified positive associations between infection load and psychiatric disorders both epidemiologically, using the random population subset of the iPSYCH sample, and genetically, using the full iPSYCH sample as well as an external replication sample, FinnGen. We have also replicated our previous result for the genetic correlation between overall infection and overall psychiatric diagnosis using the FinnGen sample. Our results also suggest that infection susceptibility could have a causal genetic relationship with psychiatric disorders. Our GWAS for infection load identified suggestive associations that were linked to genes involved in neurodevelopmental or inflammatory diseases or immune-related phenotypes. Our study thus provides further support to the notion of links between the immune system and psychiatric disorders.

## Data Availability

The iPSYCH initiative is committed to providing access to these data to the scientific community, in accordance with Danish law. Researchers may be granted access upon request to the iPSYCH management.

## Supplementary material

For supplementary material accompanying this paper visit http://doi.org/10.1017/S0950268823000687. click here to view supplementary material
