# Supplementary material for: Genetic and epidemiological analyses of infection load and its relationship with psychiatric disorders
Source: Epidemiol Infect. 2023 May 18;151:e93. doi: 10.1017/S0950268823000687 (PMC10311684; doi:10.1017/S0950268823000687)
Supplement: Supplementary file 1 [file S0950268823000687sup001.zip › S0950268823000687sup005.pdf]

Supplementary Notes for Nudel et al.: Genetic and epidemiological analyses of infection load and its relationship with psychiatric disorders

Results for LDSC analyses without the MHC region for analyses using the iPSYCH-generated LD scores. “Observed scale” refers only to binary traits; in all cases the h^2 is the one reported by LDSC.

Trait	Original		Without the MHC region	
	Observed scale h^2	SE	Observed scale h^2	SE
iPSYCH infection load (with covariate for overall psychiatric diagnosis)	0.0221	0.0055	0.0224	0.0056
iPSYCH infection load (without covariate for overall psychiatric diagnosis)	0.0252	0.0055	0.0258	0.0056
iPSYCH overall infection (with covariate for overall psychiatric diagnosis)	0.0157	0.0048	0.0154	0.0050
iPSYCH overall infection (without covariate for overall psychiatric diagnosis)	0.0178	0.0048	0.0178	0.0050
iPSYCH overall psychiatric diagnosis	0.0763	0.0061	0.0783	0.0058
FinnGen infections (iPSYCH LD scores)	0.0118	0.0030	0.0085	0.0012
FinnGen any mental disorder (iPSYCH LD scores)	0.0260	0.0019	0.0258	0.0018

Trait 1	Trait 2	Original		Without the MHC region	
		r_g	SE	r_g	SE
iPSYCH overall infection	iPSYCH overall psychiatric diagnosis	0.4061	0.1049	0.4294	0.1137
iPSYCH overall infection	FinnGen any mental disorder	0.4131	0.0956	0.3991	0.1024
iPSYCH overall infection	FinnGen infections	0.6189	0.1394	0.6978	0.1624
iPSYCH overall psychiatric diagnosis	FinnGen any mental disorder	0.6660	0.0444	0.6679	0.0487
iPSYCH overall psychiatric diagnosis	FinnGen infections	0.4062	0.1118	0.5263	0.0708
iPSYCH infection load	iPSYCH overall psychiatric diagnosis	0.4298	0.0901	0.4475	0.0912
iPSYCH infection load	FinnGen infections	0.6347	0.1279	0.7293	0.1274
iPSYCH infection load	FinnGen any mental disorder	0.3388	0.0733	0.3163	0.0743
iPSYCH infection load	iPSYCH overall infection	0.9837	0.0364	0.9840	0.0376
FinnGen infections (iPSYCH LD scores)	FinnGen any mental disorder (iPSYCH LD scores)	0.5603	0.1110	0.6989	0.0650

LCV results for genetic causality of overall infection on overall psychiatric diagnosis: GCP=0.73 ($P=4.88\times10^{-5}$). For markers with MAF≥0.05: GCP=0.79 ($P=5.21\times10^{-8}$).

Trait	Original		Without the MHC region	
	Observed scale h^2	SE	Observed scale h^2	SE
iPSYCH ASD with a history of maternal pregnancy-related infections	0.0409	0.0113	0.0410	0.0118
iPSYCH ASD with no history of maternal infections	0.0869	0.0117	0.0847	0.0126
Case-case GWAS between iPSYCH ASD with a history of maternal pregnancy-related infections, and iPSYCH ASD with no history of maternal infections	0.1059	0.0313	0.1084	0.0345
iPSYCH ASD with maternal infections occurring only more than two months after childbirth	0.0278	0.0112	0.0244	0.0113
iPSYCH ASD irrespective of maternal infections	0.1069	0.0101	0.1067	0.0107
Case-case GWAS between iPSYCH ASD with maternal infections occurring only more than two months after childbirth, and iPSYCH ASD with no history of maternal infections	-0.0203	0.0323	-0.0257	0.0334

Trait 1	Trait 2	Original		Without the MHC region	
		r_g	SE	r_g	SE
iPSYCH ASD with a history of maternal pregnancy-related infections	iPSYCH ASD with no history of maternal infections	0.3811	0.1299	0.3725	0.1403
iPSYCH ASD with a history of maternal pregnancy-related infections	PGC ASD phenotype	0.3997	0.1552	0.4508	0.1703
iPSYCH ASD with no history of maternal infections	PGC ASD phenotype	0.6735	0.0944	0.6732	0.1014
iPSYCH ASD irrespective of maternal infections	PGC ASD phenotype	0.6344	0.0830	0.6445	0.0861
iPSYCH ASD with maternal infections occurring only more than two months after childbirth	iPSYCH ASD with no history of maternal infections	1.1199	0.2565	1.1867	0.3112
iPSYCH ASD with maternal infections occurring only more than two months after childbirth	PGC ASD phenotype	0.6293	0.2134	0.7169	0.2570
iPSYCH ASD irrespective of maternal infections	iPSYCH overall infection	-0.0126	0.1061	0.0168	0.1162
