# Supplementary figures and images for: Genetic and epidemiological analyses of infection load and its relationship with psychiatric disorders
Source: Epidemiol Infect. 2023 May 18;151:e93. doi: 10.1017/S0950268823000687 (PMC10311684; doi:10.1017/S0950268823000687)

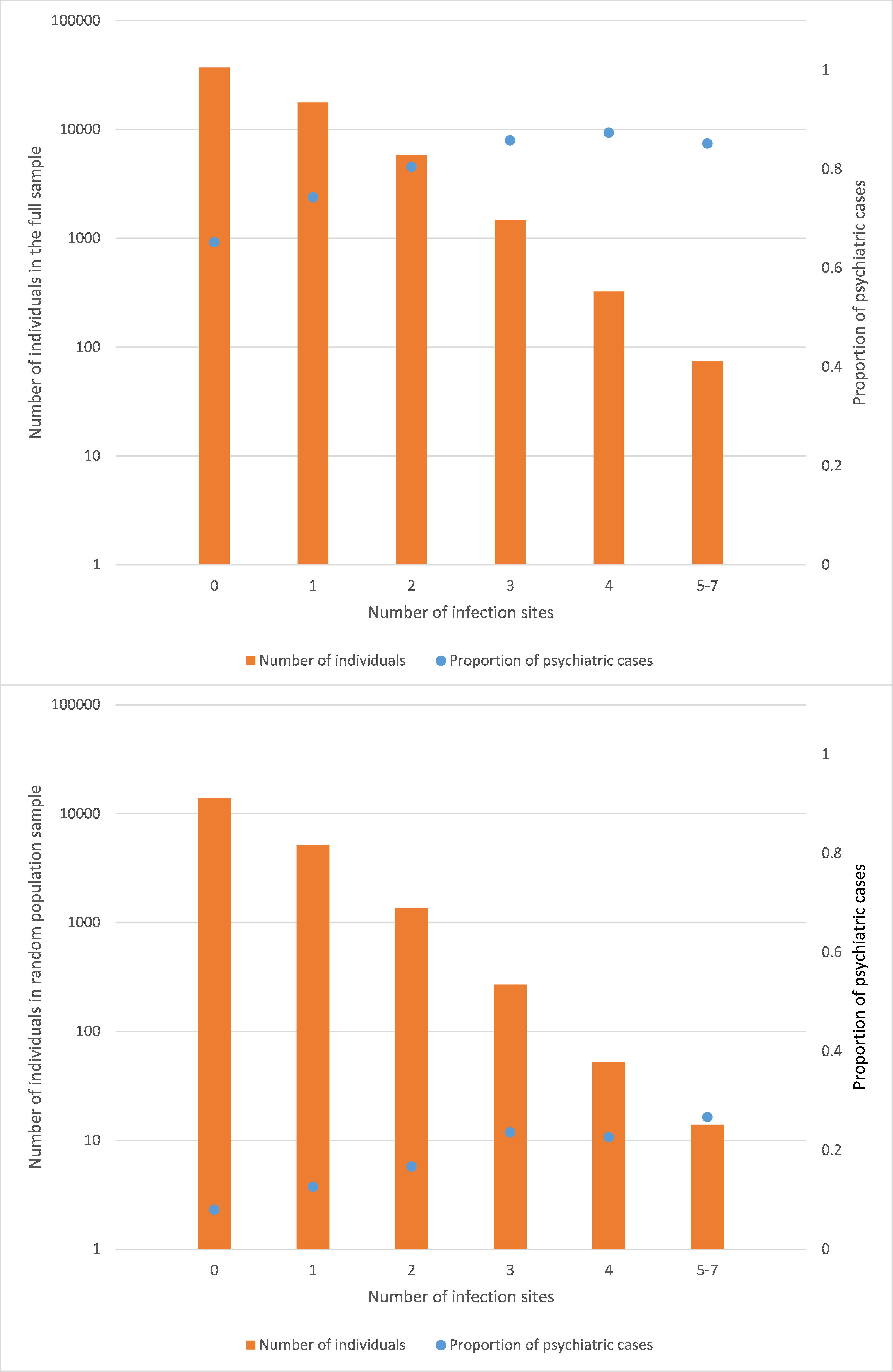

Supplement: Supplementary file 1 [file S0950268823000687sup001.zip › S0950268823000687sup001.tif]

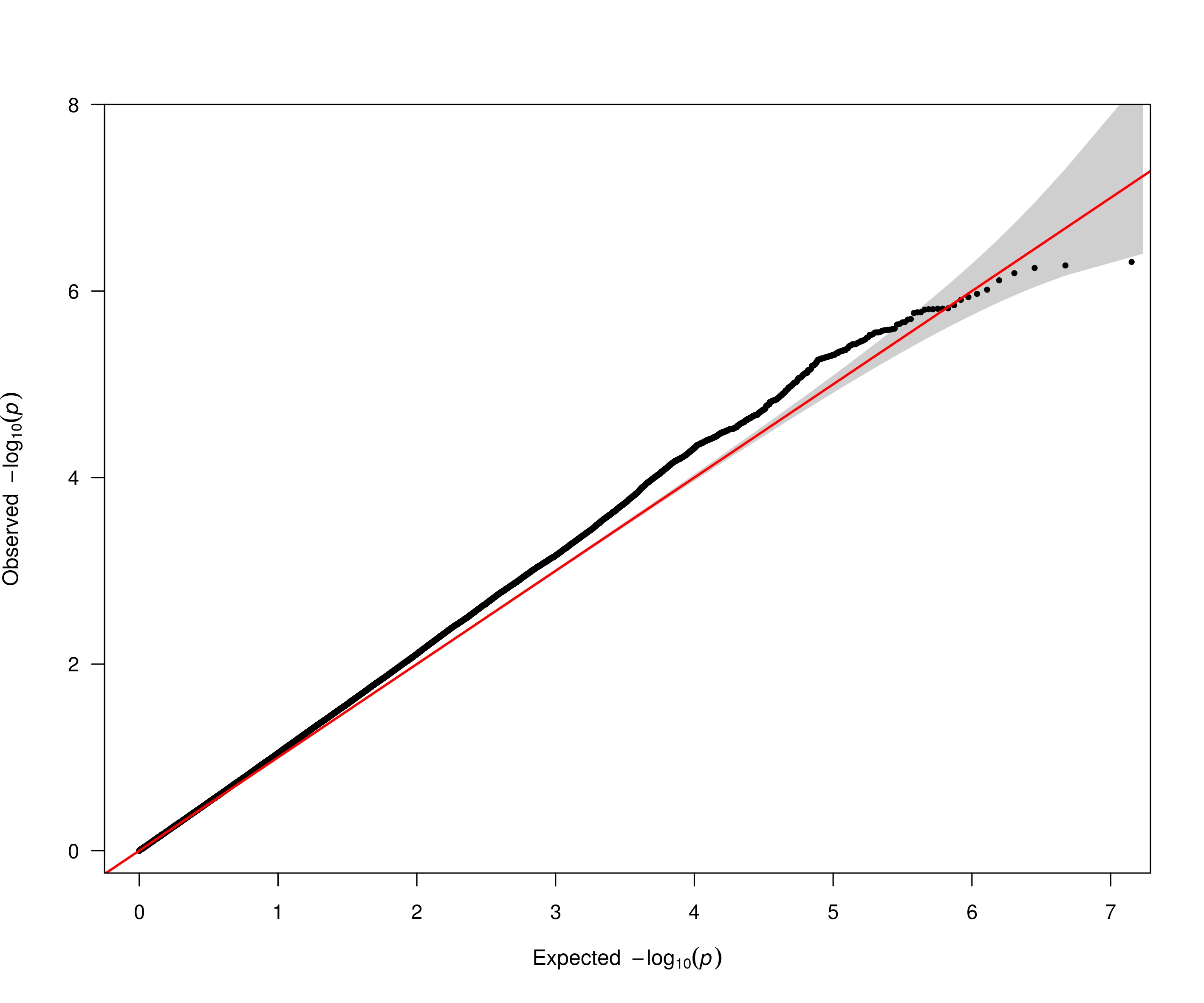

Supplement: Supplementary file 1 [file S0950268823000687sup001.zip › S0950268823000687sup002.jpg]
